# A cross-sectional study of 329 farms in England to identify risk factors for ovine clinical mastitis

**DOI:** 10.1016/j.prevetmed.2016.01.012

**Published:** 2016-03-01

**Authors:** S. Cooper, S.J. Huntley, R. Crump, F. Lovatt, L.E. Green

**Affiliations:** a School of Life Sciences, University of Warwick, Coventry CV4 7AL, England, United Kingdom; b School of Veterinary Medicine and Science, University of Nottingham, Sutton Bonington Campus, Leicestershire LE12 5RD, England, United Kingdom

**Keywords:** Ovine mastitis, Poisson regression, Incidence rate, Risk factor

## Abstract

The aims of this study were to estimate the incidence rate of clinical mastitis (IRCM) and identify risk factors for clinical mastitis in suckler ewes to generate hypotheses for future study. A postal questionnaire was sent to 999 randomly selected English sheep farmers in 2010 to gather data on farmer reported IRCM and flock management practices for the calendar year 2009, of which 329 provided usable information. The mean IRCM per flock was 1.2/100 ewes/year (CI:1.10:1.35). The IRCM was 2.0, 0.9 and 1.3/100 ewes/year for flocks that lambed indoors, outdoors and a combination of both, respectively.

Farmers ran a variety of managements before, during and after lambing that were not comparable within one model, therefore six mixed effects over-dispersed Poisson regression models were developed.

Factors significantly associated with increased IRCM were increasing percentage of the flock with poor udder conformation, increasing mean number of lambs reared/ewe and when some or all ewes lambed in barns compared with outdoors (Model 1).

For ewes housed in barns before lambing (Model 2), concrete, earth and other materials were associated with an increase in IRCM compared with hardcore floors (an aggregate of broken bricks and stones). For ewes in barns during lambing (Model 3), an increase in IRCM was associated with concrete compared with hardcore flooring and where bedding was stored covered outdoors or in a building compared with bedding stored outdoors uncovered. For ewes in barns after lambing (Model 4), increased IRCM was associated with earth compared with hardcore floors, and when fresh bedding was added once per week compared with at a frequency of ≤2 days or twice/week.

The IRCM was lower for flocks where some or all ewes remained in the same fields before, during and after lambing compared with flocks that did not (Model 5). Where ewes and lambs were turned outdoors after lambing (Model 6), the IRCM increased as the age of the oldest lambs at turnout increased.

We conclude that the reported IRCM is low but highly variable and that the complexity of management of sheep around lambing limits the insight into generating hypotheses at flock level for risks for clinical mastitis across the whole industry. Whilst indoor production was generally associated with an increased IRCM, for ewes with large litter size indoor lambing was protective, we hypothesise that this is possibly because of better nutrition or reduced exposure to poor weather and factors associated with hygiene.

## 1 Introduction

Mastitis is an inflammation of the mammary gland typically caused by bacterial infection (Khan and Khan, 2006). In suckler ewes (ewes rearing lambs for meat), clinical mastitis may be acute, with signs of local or systemic disease such as a hot or cold mammary gland, change in gait, not eating supplementary food; or chronic, when intramammary masses are detected by palpation during routine checks e.g. at weaning or before mating.

Clinical and sub-clinical mastitis result in direct and indirect economic losses for the suckler sheep industry. Costs arise from ewe and lamb deaths, culling chronically diseased ewes (Conington et al., 2008), ewe replacements and decreased live-weight gain in lambs reared by affected ewes (Fthenakis and Jones, 1990, Keisler et al., 1992, Saratsis et al., 1998, Huntley et al., 2012). An accurate estimate for the cost of mastitis to the UK sheep industry across all breeds is not available, however, a model in Texel flocks indicated that reducing the risk of mastitis by 10% would save £8.40 per ewe (Conington et al., 2008).

An estimate of the incidence rate of clinical mastitis (IRCM) depends on a farmer’s ability to detect (frequency and attentiveness of observations) and record clinical cases of mastitis. There are no estimates of the IRCM of suckler ewes in the UK. The only available estimate outside the UK is from Canada, where it was estimated to be 1.2% p.a. (0–6.6%) (Arsenault et al., 2008).

In suckler sheep, clinical cases of mastitis have been reported to peak in the first week post-partum. A second peak has been reported at 3–4 weeks of lactation in Norway (Mørk et al., 2007) and at 4–7 weeks of lactation in Ireland (Onnasch, 2000).

In dairy cows, the peak IRCM is also in the first week of lactation (Olde Riekerink et al., 2008, Waller et al., 2009). One explanation for this is that there is a pre-existing bacterial infection in the mammary gland that develops into clinical disease after the onset of lactation (Bradley and Green, 2000). Sheep also have bacteria present in the mammary gland without signs of disease (Huntley et al., 2012). As a consequence, risks for infection might not be closely related temporally to disease events, however, risks that trigger disease might be temporally close to the disease event, for example a change in ewe physiology such as the onset of lactation (Oliver and Sordillo, 1988, Kehrli et al., 1989) or the environment, such as housing. Alternatively, new bacterial infections might occur in the first week of lactation due to the opening of the teat orifice and contamination from the environment or from lambs sucking and cross-sucking, transmitting bacteria from udder skin or between ewes into the gland.

Several studies outside the UK have identified risk factors associated with mastitis in suckler ewes. Risks included litter size, breed, udder conformation, pasture type, lamb growth rate, assistance at lambing, whether the ewe had mastitis in a previous lactation, ewe age, geographical region and ewe body condition (Gross et al., 1978, Watkins et al., 1991, Larsgard and Vaabenoe, 1993, Lafi et al., 1998, Arsenault et al., 2008, Waage and Vatn, 2008). In the UK, poor udder conformation and age have been associated with high somatic cell count in individual ewes (Huntley et al., 2012).

The aims of the current study were to estimate the incidence rate of clinical mastitis and generate hypotheses for potential flock management risk factors associated with clinical mastitis, using a retrospective cross-sectional postal study of a random sample of English sheep farmers.

## 2 Materials and methods

### 2.1 Study population

The number of sheep holdings in England in the 2003 census was 45,801 (DEFRA, 2003). Based on this, a sample size of 315 flocks was required, assuming 75% of flocks had at least one case of clinical mastitis, with 95% confidence and 80% power (Win-Episcope-2, 2010). Assuming a response rate of 30% (Kaler and Green, 2008), 999 farmers whose details were provided by AHDB Beef & Lamb (formerly EBLEX), the levy body for English sheep and beef farmers, were contacted in January 2010.

### 2.2 Design of the questionnaire

Published literature and veterinary expertise on risk factors for mastitis in sheep and cattle were used to design a postal questionnaire. Questions were based on the farm, flock, ewes, management regimes, mammary gland health, nutrition and housing. There were a total of 114 questions. The majority of questions were closed or semi-closed, however, there were some open questions. These included whether farmers thought certain fields were a risk for mastitis, whether the farmer had changed farm management between 2008 and 2009 and farmer opinions on the causes of mastitis and preventive actions.

### 2.3 Pilot study

The pilot questionnaire was sent to 12 convenience selected farmers with between 50 and 1000 ewes in the north of England that included commercial and pedigree flocks situated in lowland, hill and upland areas. As a result of the feedback from the pilot study several additional questions were added to the questionnaire, and questions that had poor response rates or were answered incorrectly were re-designed.

### 2.4 Data collection & storage

The final questionnaire was sent out on 8th January 2010. A reminder was sent to non-respondents on 10th February 2010 and a second reminder and a second copy of the questionnaire were sent to non-respondents on 21st April 2010.

**Fig. 1:**
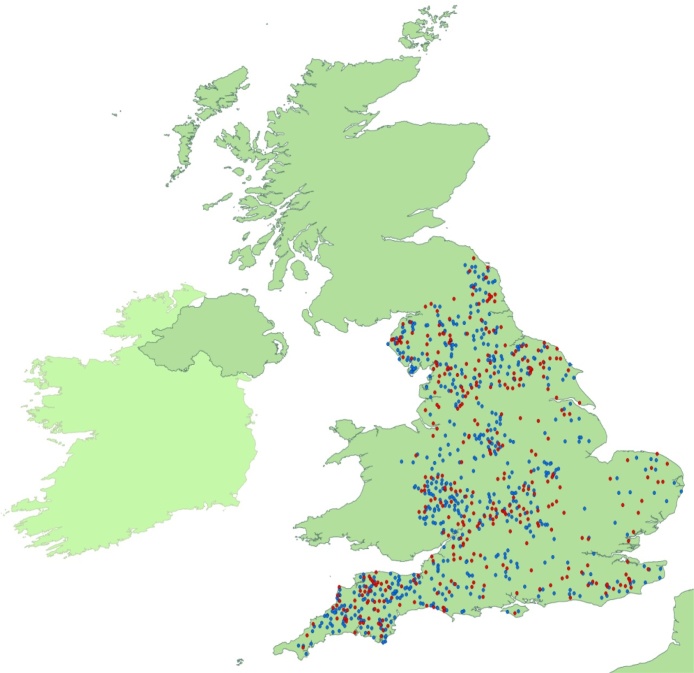
The location in England of 329 respondents (red) and non-respondents (blue) to the questionnaire. (For interpretation of the references to colour in this figure legend, the reader is referred to the web version of this article.)

A database was designed in Microsoft Access 2007. Data were entered using multiple-choice drop down boxes. The postcodes from the 999 farmers were transformed into X and Y co-ordinates and inputted into ArcView with the worldwide shapefile from the Economic and Social Research Institute (ESRI) to create a map of respondents and non-respondents (Fig. 1).

### 2.5 Data analysis

Measures of dispersion and central tendency were used to investigate the data (R Core Team, 2013). Normality was tested using Shapiro–Wilks test and the arithmetic or geometric mean was calculated for variables in *R*. Obvious errors were corrected, and categories within variables with <5 responses were merged where logical. Queries were used to select and link data from related databases in Microsoft Access for statistical analysis. Respondents with ≤20 ewes in their flock were removed from the analysis (*n* = 4). Analysis of variance (ANOVA) was used to test the differences between group means in *R*.

The incidence rate of clinical mastitis (IRCM) per flock was calculated. The variance was greater than the mean and so over-dispersed Poisson regression models, offset by flock size, were used to investigate factors associated with IRCM. A total of 144 variables were used to investigate management from 8 weeks before lambing, during lambing and during lactation. Farmers managed sheep either wholly indoors or outdoors or a combination of both, as a consequence 6 separate models were necessary. Model 1 included all respondents and covered general information about the farm, flock, lambing, mastitis, health management and nutrition. Model 2 included flocks housed in barns from up to 8 weeks before lambing to lambing. Model 3 included flocks housed during lambing, and Model 4 included flocks housed after lambing. Model 5 included flocks outdoors during lambing, and Model 6 included flocks outdoors after lambing. The percentage of flock with poor udder conformation was forced into Models 2–6. A forward stepwise approach was used and significance was determined using Wald’s test such that variables where 95% confidence intervals did not include unity were significant (*p* < 0.05).

Outliers were assessed to determine their impact on the coefficients.

The models took the following general form:

$$g(E(Y))=\beta_{0}+\sum_{}^{}B_{m}x_{m}-\log(O_{i})$$

where *g* is the log-link function, *E*(*Y*) the expected values of the outcome variable *Y* (the number of ewes with clinical mastitis in 2009), *β*_0_ the intercept and *β_m_* the regression coefficients (expressing effects of the included predictor variables *x_m_*) and *O_i_* the offset (the number of ewes in the breeding flock in 2009).

## 3 Results

### 3.1 Descriptive analysis

Of the 999 questionnaires sent out, 372 were returned (37.2% response rate), 329 of which were usable. The remaining analysis only included the 329 respondents who gave a response to both the number of ewes with clinical mastitis in 2009 and the number of breeding ewes in the flock in 2009.

### 3.2 Farm characteristics

All flocks were located in England (Fig. 1). The majority of flocks were lowland (90.3%) and commercial (66.6%). The geometric mean number of breeding ewes/flock was 248.7 (S.E = 24.8, range 21–4252). Flocks were mostly comprised of ewes that were between 2 and 5 years of age. The geometric mean number of lambs reared per ewe across all flocks was 1.2 (S.E = 0.3).

### 3.3 Incidence rate of clinical mastitis

**Fig. 2:**
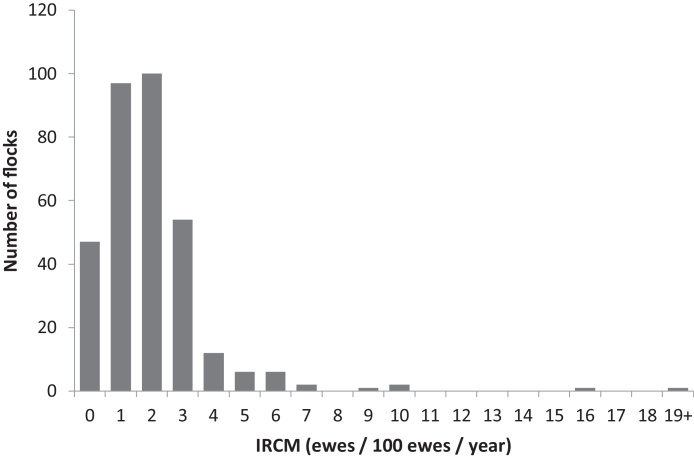
The number of flocks by incidence rate of clinical mastitis (IRCM) in England (*n* = 329).

The geometric mean IRCM across all flocks was 1.2/100 ewes/year (range = 0.0–19.0, CI: 1.10:1.35). Only 14.3% of respondents had no ewes with clinical mastitis in their flock (Fig. 2). The mean IRCM was 1.98 for ewes always housed indoors, 0.87 for ewes outdoors and 1.32 for ewes that were both outdoors and indoors; flocks that were always housed had significantly higher (*p* < 0.01) IRCM than flocks that were never housed (Table 1).

### 3.4 Clinical mastitis

**Fig. 3:**
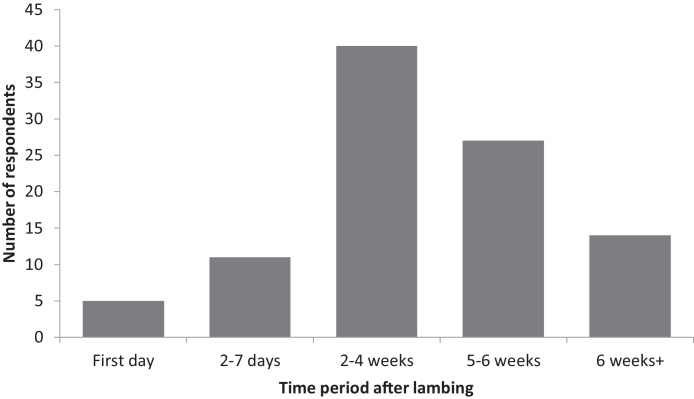
Peak in mastitis cases by time period after lambing as reported by 91 respondents.

Ninety-one respondents (27.7%) reported a peak in mastitis cases. Some respondents (*n* = 19) reported more than one peak. Respondents most frequently saw a peaks 2–4 weeks after lambing (Fig. 3).

Approximately 50% of farmers reported that all ewes that had mastitis no longer lactated from that gland whilst 9% reported that no ewes lost function in the affected gland; the remaining 41% reported that some ewes which had had clinical mastitis stopped lactating but not all. The geometric mean percentage of the flock that died due to clinical mastitis in 2009 was 0.2% (*n* = 268, CI: 0.17:0.24). On average, 3.1% (CI: 2.37:3.87) of ewes that had mastitis died. The majority of respondents did not retain ewes that had had mastitis (94.2%, *n* = 180) and did not breed from ewes which had had clinical mastitis (92.4%, *n* = 266). The most popular method of treatment of clinical mastitis was antibiotic injection (92.1%, *n* = 278), 33.1% (*n* = 100) used intramammary antibiotics and 18.2% (*n* = 55) used an anti-inflammatory treatment.

### 3.5 Management practices

**Fig. 4:**
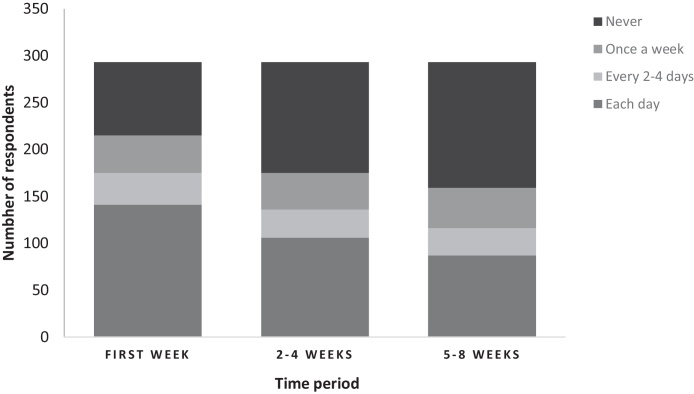
The frequency at which respondents (*n* = 293) checked ewes’ udders in the first week, 2–4 weeks and 5–8 weeks after lambing.

A total of 88.0% (287/326) of respondents checked ewes’ udders for function, disease and/or abnormalities at lambing. Less than 50.0% of respondents stated that they checked ewes specifically for mastitis at lambing. There were some respondents that never checked ewes for mastitis. Farmers were less likely to check udders as the time in lactation increased (Fig. 4).

### 3.6 Mammary gland abnormalities

Of those respondents that checked the udder at lambing, the geometric mean percentage of ewes with teat lesions or at least one occluded teat was 0.9% and 0.8% respectively. The geometric mean percent of ewes per flock with poor udder conformation was 1.3% (CI: 1.17:1.54).

Within the mastitis section of the questionnaire, there were questions about the numbers of ewes with mammary gland abnormalities, and the proportion of these ewes that were culled before tupping and at weaning (Table 2). Of the 281 respondents that had at least one ewe with mastitis between weaning 2008 and tupping 2009, 127 completed this section. On average, there was a slightly higher percentage of the flock with mammary gland abnormalities at weaning compared with before tupping (Table 2), except for ewes with teat cords (hard fibrous structure identified by palpating the teat), where more of these abnormalities were observed before tupping than at weaning (Table 2). Respondents managed different abnormalities differently; for example respondents culled on average between 85.6% and 93.1% of ewes with clinical mastitis at weaning and before tupping respectively whereas a smaller percentage of ewes affected with teat warts were culled at weaning and before tupping (31.8% and 29.9% respectively) (Table 2).

### 3.7 Risk factors for clinical mastitis

The significant (*p* < 0.05) univariable associations between exposures and IRCM are presented in Table 3, Table 4. The variables in each multivariable model are presented below.

#### 3.7.1 Model 1—general flock management

As the percentage of the flock with poor udder conformation increased, the IRCM increased (RR: 1.11; CI: 1.05:1.16) (Table 5). Lambing some (RR: 0.28; CI: 0.09:0.89) or all ewes (RR: 0.01; CI: 0.00:0.18) outdoors was associated with a decreased IRCM compared with lambing all ewes indoors (Table 1, Table 5). There was an interaction between the number of lambs reared per ewe and ewes lambed indoors with respect to IRCM; for flocks that lambed indoors, as the number of lambs per ewe increased, the IRCM decreased. Conversely, for flocks that were lambed partly or wholly outdoors, as the number of lambs per ewe increased the IRCM also increased (RR: 2.48, CI: 1.13:5.44, RR: 13.89; CI: 2.14:90.03) (Table 5).

#### 3.7.2 Model 2—ewes housed in barns before lambing

The base material of the floor in the barn before lambing was significantly associated with the IRCM; concrete (RR: 1.56; CI: 1.09:2.22), earth (RR: 1.55; CI: 1.07:2.24) and other materials (RR: 1.82; CI: 1.15:2.90) were associated with an increase in IRCM compared with a base material of hardcore (Table 6).

#### 3.7.3 Model 3—ewes housed during lambing

The base material of the floor in the barn at lambing was also significantly associated with the IRCM: concrete based floors (RR: 1.87; CI: 1.11:3.13) were associated with an increase in the IRCM compared with a base material of hardcore (Table 6). There was an increase in IRCM when bedding was stored covered outdoors (RR: 2.54; CI: 1.09:5.96) or in a building (RR: 1.34; CI: 0.72:2.48) compared with bedding stored outdoors uncovered (Table 6).

#### 3.7.4 Model 4—ewes housed after lambing

Earth (RR: 2.59; CI: 1.25:5.38) flooring was associated with an increase in IRCM compared with a base material of hardcore (Table 6). The IRCM was lower in flocks where fresh bedding was added ≤2 days (RR: 0.52; CI: 0.23:1.16), or twice each week (RR: 0.52; CI: 0.39:0.93) compared with flocks where fresh bedding was added weekly.

#### 3.7.5 Model 5—ewes that lambed outdoors

Flocks with some (RR: 0.40; CI: 0.22:0.73) or all (RR: 0.55; CI: 0.32:0.94) ewes kept in the same field before, during and after lambing had a decreased IRCM, compared with those where no ewes remained in the same field before, during and after lambing (Table 7).

#### 3.7.6 Model 6—ewes reared outdoors after lambing

The IRCM increased as the age of the oldest lambs at turnout increased (RR: 1.01; CI: 1.00:1.02) (*P* < 0.05) for each extra day the ewe and lambs were housed before turnout.

## 4 Discussion

This is the first study to estimate the IRCM and to identify flock level managements and risk factors for clinical mastitis in suckler sheep from a random sample of farmers in England. Farmers used a range of managements from late pregnancy to weaning that resulted in comparisons between sub-groups of farmers rather than one model for the whole dataset. Risk factors associated with higher IRCM included udder conformation, litter size, use of housing/pasture, floor materials and management of straw bedding. These are discussed below.

The percentage of ewes within a flock with poor udder conformation was significantly positively associated with IRCM. Whilst poor udder conformation was not defined in the questionnaire, and we cannot be certain what farmers considered as poor conformation, this association was also detected in a longitudinal study of 67 suckler ewes (Huntley et al., 2012). In that study pendulous udders, extreme teat positions and greater cross-sectional area of the teats were aspects of udder conformation associated with an increase in somatic cell count, indicative of subclinical infection (Huntley et al., 2012). The results from both that and the current study suggest that such ewes are at risk of clinical mastitis compared with ewes with normal udder conformation and that this is detectable at flock as well as individual level.

The IRCM in flocks that were always housed was significantly higher than that in flocks that were always outdoors, and indeed any period of housing was associated with a higher IRCM. In addition, the IRCM increased as the age of the oldest lambs at turnout increased. This is a measure of the time ewes and lambs were housed after lambing; the longer ewes and lambs were kept indoors, the higher the risk of clinical mastitis. One explanation for the increased IRCM and housing is that stocking density in housed ewes is higher than the stocking density of ewes at pasture. There is an increased bacterial load with increased stocking density (Sevi et al., 1999) both in pasture and housed conditions. In the housed environment bacterial contamination would be exacerbated by contaminated straw; in the current study, bedding stored covered outdoors, where it might become warm and damp enabling bacterial growth, was associated with higher IRCM. Contact with bacteria can be reduced by a layer of fresh straw and a faster rate of addition of fresh straw was associated with a lower IRCM in our study. Deep straw bedding at calving was negatively correlated with IRCM in a study of 274 dairy cow herds (Barkema et al., 1999).

In the current study, concrete, earth and other materials were associated with an increase in IRCM compared with hardcore at various stages of housing. Several studies of other ruminants have identified associations between floor type and mastitis. There was an increase in the detection of bacteria in milk in a longitudinal study of 315 dairy goats housed on earth floors compared with does housed on raised timber floors (Ndegwa et al., 2000). In a study of 245 dairy cows in southern Ethiopia, cows in houses with soil floors had a higher IRCM than cows on concrete floors (Abera et al., 2012). In a cross-sectional study of 1923 dairy cattle farms by Ruud et al. (2010), the IRCM decreased on flooring materials such as rubber, multilayer mats and mattresses compared with concrete (Ruud et al., 2010). The association between floor type and the infection status of an animal is probably linked to how easy it is for a floor to be cleaned. In our study, hardcore flooring resulted in a decrease in IRCM compared to other floor types. This seems surprising but could be because hardcore, being more uneven than the other floor types, allows for better drainage of fluids reducing the likelihood of bedding becoming moist, which would aid bacterial colonisation. Alternatively, it could be that bedding depth is greater to protect ewes from the hardcore. This finding warrants further investigation.

Several studies have shown ewes rearing more than one lamb are more susceptible to mastitis than ewes rearing a single lamb (Gross et al., 1978, Watkins et al., 1991, Larsgard and Vaabenoe, 1993, Arsenault et al., 2008, Waage and Vatn, 2008). In the current study, ewes with more than one lamb were less likely to get mastitis when housed and more likely to get mastitis when at pasture. This difference is likely to be because farmers have different management practices for ewes with twins or triplets by system. For example, housed ewes with twins or triplets might be fed more and checked more regularly than ewes with multiple lambs outdoors where they might not have access to supplementary feed or be competing with ewes with a single lamb, this would increase cumulative stress on the udder which might result in mastitis.

Previous studies have identified breed, ewe age, geographical region and ewe body condition as risk factors for mastitis (Watkins et al., 1991, Arsenault et al., 2008, Waage and Vatn, 2008). In the current study none of these risks was significantly associated with flock mean IRCM. Ewe age and body condition are probably distributed similarly within flocks and so this greater risk of mastitis is not detected at flock level. In the case of geographical region, this could be because the previous studies were undertaken outside the UK where the environment (such as weather conditions, vegetation, topology and soil composition) vary more by geographical region, unlike England where climate and topography is less variable.

As well as the risks for IRCM, we requested information on farmer management of ewes around lambing. Only 87.2% of respondents checked ewes’ udders at lambing, and the regularity of these checks reduced rapidly over subsequent weeks. The regularity of udder checks might also be affected by differences in management practices. It is possible that farmers with indoor flocks were able to check ewes more frequently and therefore the detection of more cases of clinical mastitis (Table 1) is an observation bias. These farmer behaviours indicate that there is likely to be under-reporting of mild cases of mastitis and other time varying factors associated with mastitis. For example, teat lesions are relatively transient in nature (Cooper et al., 2013) compared with udder conformation that is less changeable over time and the irregularity of farmer checks coupled with the transient nature of teat lesions might have resulted in an under-estimation of teat lesions, and therefore an inaccurate identification of a lack of association with flock mean IRCM.

There are many challenges to postal questionnaires, including responder bias, under-estimation of disease prevalence, external validity, ensuring respondents understand questions and low response rates which could alter the results of a study. In the current study, the design of some questions may have resulted in lack of association between explanatory variables and the IRCM. For example in this study, the categories available for body condition of ewes were ‘too thin’, ‘about right’ or ‘too fat’. Most respondents thought their ewes were ‘about right’. As discussed above, flock level body condition might not be associated with IRCM, however, another possibility is that the three categories might not have been sufficient to discern such a relationship at flock level or, as above, this would be a within flock rather than between flock risk.

Postal questionnaires depend on the respondents’ willingness to participate. Selective non-response might lead to bias in the prevalence of disease (Hoeymans et al., 1998, Hardie et al., 2003). In this study, respondents may have had a different IRCM than non-respondents resulting in an under or over-estimation in IRCM. The geographical locations of both respondents and non-respondents were similar (Fig. 1), and therefore non-response did not appear to be affected by geographical location. There was no information on flock size of non-respondents and so this cannot be assessed.

Lack of responses to individual questions reduces power and the probability of detecting true significant associations. For example, nutrition is associated with IRCM in dairy cow herds (Barkema et al., 1999). In the current study, although nutrition was investigated, there were low response rates to these questions, and therefore they could not be included in the Poisson regression models. In a recent on-farm longitudinal study (Grant et al., unpublished) inadequate nutrition, both protein and energy, were associated with the risk of clinical mastitis.

This was a hypothesis generating study, and variables need to be tested further in longitudinal studies to confirm statistical associations as well as the direction of these associations. From the current study, environmental hygiene and host susceptibility (indicated by udder conformation) are avenues to investigate further.

## 5 Conclusions

The mean flock incidence rate of clinical mastitis was 1.2/100 ewes/year in 372 randomly selected sheep flocks in England. Possible risk factors for clinical mastitis included udder conformation and rearing single or multiple lambs. Indoor management before, during and after lambing was associated with increased risk of IRCM, however, indoor lambing appeared to protect ewes with larger litter sizes. Whilst the majority of farmers checked ewes’ udders after lambing, not all did this management and checking the udder was rarely done after 5 weeks into lactation. Some risk factors identified in the current study have been identified previously, whereas others, such as floor type and storage of bedding, are novel and warrant further research.

## Conflict of interest

The authors have no conflicts of interest to declare.

## Figures and Tables

**Table 1:** The mean incidence rate of clinical mastitis (IRCM) by stage of production and ewe location.

		IRCM (no. ewes/100 ewes/year)			
		Stage of production			
		Before lambing	At lambing	After lambing	Overall
Ewe location	Outdoor	0.87	0.82	0.99	0.87
Both	NA	1.25	1.41	1.32
Indoors	1.39	1.41	1.82	1.98

**Table 2:** The mean percentage of flock affected and culled with different mammary gland abnormalities.

	Mean percentage of flock affected with abnormality		Mean percentage of flock culled with abnormality		Mean percentage of affected ewes culled	
	Before tupping	Weaning	Before tupping	Weaning	Before tupping	Weaning
Mass in mammary gland	0.55	0.68	0.50	0.60	90.87	87.58
Clinical mastitis	0.22	0.34	0.20	0.29	93.11	85.58
Teat cord	0.16	0.12	0.13	0.10	81.59	79.44
Teat skin damage	0.04	0.05	0.02	0.02	43.72	50.33
Teat warts	0.09	0.14	0.03	0.05	29.94	31.79

**Table 3:** Significant (*p* < 0.05) univariable over-dispersed Poisson regression analyses for continuous explanatory variables on farm and flock, lambing, health and feeding management associated with incidence rate of clinical mastitis.

Variable		*N*	RR	Lower CI	Upper CI
Percentage of flock with mastitis in previous year kept for breeding in 2009		260	1.62	1.26	2.08
Percentage of flock with poor udder conformation		173	1.13	1.08	1.18
Percentage of flock ‘too fat’ versus ‘about right’	At tupping	293	1.01	1.00	1.01
Mid pregnancy	270	1.01	1.00	1.02
Mid lambing	285	1.01	1.00	1.02
Percentage of flock with singles at scanning		128	0.98	0.97	0.99
The percentage of flock with twins at scanning		129	0.99	0.98	1.01
The percentage of flock with triplets at scanning		127	1.04	1.02	1.06
The percentage of flock with poor udder conformation of the respondents that checked the mammary gland of ewes at lambing		247	1.05	1.01	1.10
The number of lambs reared per ewe		271	0.95	0.74	1.23
Percentage of lambs finished before weaning		294	1.00	1.00	1.01
Percentage of flock with mastitis kept to breed		288	1.62	1.26	2.08
Percentage of flock with poor udder conformation		173	1.13	1.08	1.18
Age of oldest lambs at turnout (days)		233	1.01	1.00	1.01

*N* = number of respondents who gave a valid response to the question. RR = risk ratio. Lower and upper CI = 96% confidence intervals for the risk ratio.

**Table 4:** Significant (*P* < 0.05) univariable over-dispersed Poisson regression analyses for categorical explanatory variables on farm and flock, lambing, health and feeding management associated with incidence rate of clinical mastitis.

Variable	Category	*N*	% with CM	IRCM	RR	Lower CI	Upper CI
Pedigree or commercial flock	Commercial	219	89.5	1.27	Reference		
Both	12	83.3	1.46	1.36	0.88	2.10
Pedigree	95	78.9	1.09	0.62	0.48	0.78
Texel	No	240	86.3	1.13	Reference		
Yes	86	86.0	1.49	1.34	1.08	1.66
Mule	No	173	80.3	1.17	Reference		
Yes	153	92.8	1.29	1.43	1.16	1.75
Replacement ewes	Home bred	149	81.2	1.14	Reference		
Both	69	91.3	1.33	1.26	0.98	1.63
Bought in	106	89.6	1.28	1.42	1.12	1.79
Location of ewes at lambing	Indoors	166	89.2	1.41	Reference		
Both	81	88.9	1.25	0.75	0.60	0.94
Outdoors	78	74.4	0.82	0.46	0.35	0.61
Location of ewes after lambing	Indoors	12	91.7	1.82	Reference		
Both	176	88.1	1.41	0.96	0.65	1.44
Outdoors	130	84.6	0.99	0.61	0.40	0.93
Location of ewes at all time points	Indoors	11	90.9	1.98	Reference		
Both/changed	242	88.8	1.32	0.86	0.57	1.29
Outdoors	62	77.4	0.87	0.42	0.26	0.69
Location of ewes before lambing	Indoors	230	89.6	1.39	Reference		
Outdoors	99	76.8	0.87	1.67	1.32	2.11
Ewes vaccinated with Covexin (Schering-Plough Animal Health Corporation, U.S.A)	No	224	89.3	1.3	Reference		
Yes	51	80.4	0.99	0.70	0.53	0.95
Ewes vaccinated with Scabivax	No	254	87	1.21	Reference		
(Mallinckrodt Veterinary Ltd., U.K.)	Yes	21	95.2	1.71	1.76	1.25	2.48
Lambs vaccinated with Heptavac	No	167	90.4	1.33	Reference		
(Hoechst Ltd., U.K.)	Yes	6	66.7	0.46	0.34	0.12	0.97
Ewes vaccinated against Mannheimia	No	54	77.8	0.99	Reference		
Yes	221	90	1.31	1.33	1.00	1.76
Proportion of ewes with mastitis treated with an anti-inflammatory	None	247	91.1	1.35	Reference		
Some	16	100	1.58	1.43	1.03	1.98
All	39	97.4	1.43	1.20	0.92	1.58

Water provision at lambing	Restricted	274	85.4	1.31	Reference		
Unlimited/river	27	92.6	0.76	0.57	0.39	0.83
Mix	26	80.8	0.80	0.49	0.35	0.70
Water provision after lambing	Restricted	235	86.0	1.35	Reference		
Unlimited/river	44	84.1	0.99	0.65	0.48	0.90
Mix	48	85.4	0.81	0.83	0.65	1.06
Frequency water was changed at lambing	Ad lib	30	82.2	0.76	Reference		
Once a day	44	87.5	1.27	1.56	1.05	2.32
Twice a day	68	89.8	1.45	1.71	1.18	2.48
Three times a day	3	80.0	1.39	1.43	0.68	2.99
Frequency water was changed after lambing where housed	Ad lib	45	86.7	0.79	Reference		
Once a day	24	94.1	1.30	1.24	0.79	1.93
Twice a day	59	92.6	1.54	1.76	1.24	2.51
Three times a day	5	66.7	1.52	1.89	0.69	5.23
How often water was topped up at lambing	Ad lib	28	92.9	0.80	Reference		
Once a day	22	86.4	1.56	1.49	0.91	2.44
Twice a day	59	94.9	1.58	1.69	1.15	2.48
Three times a day	14	64.3	1.10	2.82	1.72	4.62
How often water was topped up after lambing	Ad lib	45	84.4	0.81	Reference		
Once a day	11	90.9	1.07	1.17	0.62	2.20
Twice a day	57	93.0	1.56	1.6	1.08	2.35
Three times a day	20	75.0	1.02	1.10	0.60	2.02
Proportion of ewes that were able to eat concentrate at one time	All of the ewes	285	88.4	1.28	Reference		
Most of the ewes	11	63.6	0.67	0.46	0.23	0.95
Less than half of the ewes	3	100.0	3.85	2.78	1.36	5.72
Age at which lambs were offered creep feed	Not offered creep	180	86.1	1.15	Reference		
Less than 1 week	14	85.7	1.60	1.07	0.60	1.91
1–3 weeks old	76	88.2	1.38	1.28	1.02	1.61
4–8 weeks old	45	80.0	1.28	1.34	0.99	1.81

Base material of the floor indoors before lambing	Hardcore	60	84.0	1.03	Reference		
Concrete	110	95.7	1.69	1.60	1.26	2.04
Earth	52	87.0	1.41	1.63	1.24	2.14
Mix or other floor types	24	85.0	1.20	1.93	1.37	2.70
Base material of the floor indoors at lambing	Hardcore	25	84.0	1.05	Reference		
Concrete	125	91.2	1.52	1.67	1.13	2.45
Earth	59	86.4	1.30	1.65	1.08	2.52
Mix or other floor types	36	88.9	1.15	1.38	0.88	2.15
How often fresh bedding was added to lambing pens at lambing	Daily or more	177	89.3	1.35	Reference		
With each ewe	33	93.0	1.52	1.23	0.97	1.56
Every few days +	6	100.0	2.77	2.12	1.26	3.58
When needed	17	88.2	1.14	0.90	0.57	1.40
Bedding storage	Outdoors uncovered	14	85.7	1.23	Reference		
Outdoors covered	14	85.7	1.62	1.99	1.05	3.78
In a building	213	89.2	1.34	1.17	0.67	2.02
Mixed	5	100.0	1.73	1.21	0.46	3.20
Whether the same housing was used for ewes and lambs before and after lambing	No	122	88.5	1.25	Reference		
Yes	104	91.3	1.57	1.28	1.04	1.59
Whether the same housing was used for ewes and lambs at and after lambing	No	83	89.2	1.60	Reference		
Yes	97	87.6	1.29	0.76	0.59	0.98
How often fresh bedding was added to lambing pens after lambing	Weekly or more	7	100.0	3.00	Reference		
Twice a week	23	87.0	1.34	0.52	0.28	0.96
Every 2 days or less	144	88.2	1.40	0.42	0.24	0.74

*N* = number of respondents who gave a valid response to the question. % = percentage of flocks with CM (clinical mastitis). RR = risk ratio. Lower and upper CI = 95% confidence intervals for the risk ratio. IRCM = incidence rate of clinical mastitis: no. ewes/100 ewes/year with clinical mastitis.

**Table 5:** Model 1—general flock management. An over-dispersed Poisson regression model of risk factors associated with the incidence rate of clinical mastitis for 148 respondents in England.

Variable		IRCM	RR	Lower CI	Upper CI
Intercept-4.14 (0.24)					
Percentage of flock with poor udder conformation			1.11	1.05	1.16
Number of lambs reared per ewe			0.76	0.54	1.07
Management at lambing	Indoors	1.41	Reference		
Both	1.25	0.28	0.09	0.89
Outdoors	0.82	0.01	0.00	0.18
Number of lambs reared per ewe × management at lambing	Indoors		Reference		
Both		2.48	1.13	5.44
Outdoors		13.89	2.14	90.03

RR = risk ratio. Lower and upper CI = 95% confidence intervals for the risk ratio. IRCM = incidence rate of clinical mastitis: no. ewes/100 ewes/year with clinical mastitis.

**Table 6:** Over-dispersed Poisson regression Models 2–4 of risk factors associated with the incidence rate of clinical mastitis. Model 2—ewes housed in barns before lambing, Model 3—ewes housed at lambing, Model 4—ewes housed after lambing.

Variable		IRCM	RR	Lower CI	Upper CI
Model 2 before lambing (*n* = 230)					
Intercept-4.82 (0.18)					
Percentage of flock with poor udder conformation			1.12	1.06	1.17
Base material of the floor	Hardcore	1.03	Reference		
Concrete	1.69	1.56	1.09	2.22
Earth	1.41	1.55	1.07	2.24
Other	1.20	1.82	1.15	2.90

Model 3 during lambing (*n* = 247)					
Intercept-5.11 (0.41)					
Percentage of flock with poor udder conformation			1.08	1.01	1.15
Base material of the floor	Hardcore	1.05	Reference		
Concrete	1.52	1.87	1.11	3.13
Earth	1.30	1.62	0.88	2.97
Other	1.15	1.53	0.79	2.95
Bedding storage	Outdoors uncovered	1.23	Reference		
Outdoors covered	1.62	2.54	1.09	5.96
In a building	1.34	1.34	0.72	2.48
Mixed	1.73	1.03	0.21	4.97

Model 4 after lambing (*n* = 187)					
Intercept-5.07 (0.33)					
Percentage of flock with poor udder conformation			1.13	1.06	1.19
Base material of the floor	Hardcore	0.88	Reference		
Concrete	1.48	1.88	0.95	3.69
Earth	1.47	2.59	1.25	5.38
Other	1.45	2.00	0.95	4.19
Frequency of adding fresh bedding	Weekly	3.00	Reference		
Twice a week	1.34	0.52	0.39	0.93
Every two days or less	1.40	0.52	0.23	1.16

RR = Risk ratio. Lower and upper CI = 95 percent confidence intervals for the risk ratio. IRCM = incidence rate of clinical mastitis: no. ewes/100 ewes/year with clinical mastitis.

**Table 7:** Over-dispersed Poisson regression Models 5 and 6 of risk factors associated with the incidence rate of clinical mastitis. Model 5—ewes that lambed outdoors, and Model 6—ewes reared outdoors after lambing.

Variable		IRCM	RR	Lower CI	Upper CI
Model 5 at lambing (*n* = 160)					
Intercept-4.36 (0.16)					
Percentage of flock with poor udder conformation			1.05	0.96	1.15
Proportion of ewes kept in the same fields before, at and after lambing	None	1.09	Reference		
Some	0.92	0.40	0.22	0.73
All	0.90	0.55	0.32	0.94

Model 6 after lambing (*n* = 306)					
Intercept-7.44 (0.61)					
Percentage of flock with poor udder conformation			1.12	1.08	1.17
Age of oldest lambs at turnout			1.01	1.00	1.02

RR = risk ratio. Lower and upper CI = 95% confidence intervals for the risk ratio. IRCM = incidence rate of clinical mastitis: no. ewes/100 ewes/year with clinical mastitis.

## References

[bib0005] Abera M., Elias B., Aragaw K., Denberga Y., Amenu K., Sheferaw D. (2012). Major causes of mastitis and associated risk factors in smallholder dairy cows in Shashemene, Southern Ethiopia. Afr. J. Agric. Res..

[bib0010] Arsenault J., Dubreuil P., Higgins R., Bélanger D. (2008). Risk factors and impacts of clinical and subclinical mastitis in commercial meat-producing sheep flocks in Quebec, Canada. Prev. Vet. Med..

[bib0015] Barkema H.W., Schukken Y.H., Lam T.J.G.M., Beiboer M.L., Benedictus G., Brand A. (1999). Management practices associated with the incidence rate of clinical mastitis. J. Dairy Sci..

[bib0020] Bradley A.J., Green M.J. (2000). A study of the incidence and significance of intramammary enterobacterial infections acquired during the dry period. J. Dairy Sci..

[bib0025] Conington J., Cao G., Stott A., Bünger L. (2008). Breeding for resistance to mastitis in United Kingdom sheep, a review and economic appraisal. Vet. Rec..

[bib0030] Cooper S., Huntley S.J., Green L.E. (2013). A longitudinal study of risk factors for teat lesions in 67 suckler ewes in a single flock in England. Prev. Vet. Med..

[bib0035] DEFRA, 2003. (Department for Environment, Food and Rural Affairs) NADIS and census estimates of coverage of livestock and holdings in England.

[bib0040] Fthenakis G.C., Jones J.E.T. (1990). The effect of experimentally induced subclinical mastitis on milk yield of ewes and on the growth of lambs. Br. Vet. J..

[bib0045] Grant C., Smith E.M., Green L.E. (2016). A Longitudinal Study of Factors Associated with Acute and Chronic Mastitis and Their Impact on Lamb Growth Rate on 10 Suckler Sheep Flocks in Great Britain.

[bib0050] Gross S.J., Pollak E.J., Anderson J.G., Torell D.T. (1978). Incidence and importance of subclinical mastitis in sheep. J. Anim. Sci..

[bib0055] Hardie J.A., Bakke P.S., Mørkve O. (2003). Non-response bias in a postal questionnaire survey on respiratory health in the old and very old. Scand. J. Public Health.

[bib0060] Hoeymans N., Feskens E.J.M., Van Den Bos G.A.M., Kromhout D. (1998). Non-response bias in a study of cardiovascular diseases, functional status and self-rated health among elderly men. Age Ageing.

[bib0065] Huntley S.J., Cooper S., Bradley A.J., Green L.E. (2012). A cohort study of the associations between udder conformation, milk somatic cell count, and lamb weight in suckler ewes. J. Dairy Sci..

[bib0070] Kaler J., Green L.E. (2008). Naming and recognition of six foot lesions of sheep using written and pictorial information: a study of 809 English sheep farmers. Prev. Vet. Med..

[bib0075] Kehrli M.E., Nonnecke B.J., Roth J.A. (1989). Alterations in bovine neutrophil function during the periparturient period. Am. J. Vet. Res..

[bib0080] Keisler D.H., Andrews M.L., Moffatt R.J. (1992). Subclinical mastitis in ewes and its effect on lamb performance. J. Anim. Sci..

[bib0085] Khan M.Z., Khan A. (2006). Basic facts of mastitis in dairy animals. Pak. Vet. J..

[bib0090] Lafi S.Q., Al-Majali A.M., Rousan M.D., Alawneh J.M. (1998). Epidemiological studies of clinical and subclinical ovine mastitis in Awassi sheep in northern Jordan. Prev. Vet. Med..

[bib0095] Larsgard A.G., Vaabenoe A. (1993). Genetic and environmental causes of variation in mastitis in sheep. Small Rumin. Res..

[bib0100] Mørk T., Waage S., Tollersrud T., Kvitle B., Sviland S. (2007). Clinical mastitis in ewes; bacteriology, epidemiology and clinical features. Acta Vet. Scand..

[bib0105] Ndegwa E.N., Mulei C.M., Munyua S.J.M. (2000). Risk factors associated with subclinical subacute mastitis in Kenyan dairy goats. Isr. J. Vet. Med..

[bib0110] Olde Riekerink R.G.M., Barkema H.W., Kelton D.F., Scholl D.T. (2008). Incidence rate of clinical mastitis on Canadian dairy farms. J. Dairy Sci..

[bib0115] Oliver S.P., Sordillo L.M. (1988). Udder health in the periparturient period. J. Dairy Sci..

[bib0120] Onnasch H. (2000). A study of mastitis in Irish sheep. MS Thesis. Faculty of Veterinary Medicine.

[bib0125] R Core Team (2013). R: A Language and Environment for Statistical Computing. arxiv:/www.R-project.org.

[bib0130] Ruud L.E., Bøe K.E., Østerås O. (2010). Associations of soft flooring materials in free stalls with milk yield, clinical mastitis, teat lesions, and removal of dairy cows. J. Dairy Sci..

[bib0135] Saratsis P., Leontides L., Tzora A., Alexopoulos C., Fthenakis G.C. (1998). Incidence risk and aetiology of mammary abnormalities in dry ewes in 10 flocks in Southern Greece. Prev. Vet. Med..

[bib0140] Sevi A., Massa S., Annicchiarico G., Dell’Aquila S., Muscio A. (1999). Effect of stocking density on ewes’ milk yield, udder health and microenvironment. J. Dairy Res..

[bib0145] Waage S., Vatn S. (2008). Individual animal risk factors for clinical mastitis in meat sheep in Norway. Prev. Vet. Med..

[bib0150] Waller K.P., Bengtsson B., Lindberg A., Nyman A., Unnerstad H.E. (2009). Incidence of mastitis and bacterial findings at clinical mastitis in Swedish primiparous cows—influence of breed and stage of lactation. Vet. Microbiol..

[bib0155] Watkins G.H., Burriel A.R., Jones J.E.T. (1991). A field investigation of subclinical mastitis in sheep in southern England. Br. Vet. J..

[bib0160] Win-Episcope-2 (2010). Computer-aided in Learning in Veterinary Education (CLIVE). http://www.clive.ed.ac.uk.

